# The *Bacillus anthracis* S-layer is an exoskeleton-like structure that imparts mechanical and osmotic stabilization to the cell wall

**DOI:** 10.1093/pnasnexus/pgac121

**Published:** 2022-08-04

**Authors:** Antonella Fioravanti, Marion Mathelie-Guinlet, Yves F Dufrêne, Han Remaut

**Affiliations:** Structural and Molecular Microbiology, Structural Biology Research Center, VIB, Pleinlaan 2, 1050 Brussels, Belgium; Structural Biology Brussels, Vrije Universiteit Brussel, Pleinlaan 2, 1050 Brussels, Belgium; Louvain Institute of Biomolecular Science and Technology, UCLouvain, Croix du Sud, 4-5, bte L7.07.07, B-1348 Louvain-la-Neuve, Belgium; Louvain Institute of Biomolecular Science and Technology, UCLouvain, Croix du Sud, 4-5, bte L7.07.07, B-1348 Louvain-la-Neuve, Belgium; Structural and Molecular Microbiology, Structural Biology Research Center, VIB, Pleinlaan 2, 1050 Brussels, Belgium; Structural Biology Brussels, Vrije Universiteit Brussel, Pleinlaan 2, 1050 Brussels, Belgium

**Keywords:** S-layer, cell envelope, bionanomechanics, *B. anthracis*, exoskeleton, force spectroscopy

## Abstract

Surface layers (S-layers) are 2D paracrystalline protein monolayers covering the cell envelope of many prokaryotes and archaea. Proposed functions include a role in cell support, as scaffolding structure, as molecular sieve, or as virulence factor. *Bacillus anthracis* holds two S-layers, composed of Sap or EA1, which interchange in early and late exponential growth phase. We previously found that acute disruption of *B. anthracis* Sap S-layer integrity, by means of nanobodies, results in severe morphological cell surface defects and cell collapse. Remarkably, this loss of function is due to the destruction of the Sap lattice structure rather than detachment of monomers from the cell surface. Here, we combine force nanoscopy and light microscopy observations to probe the contribution of the S-layer to the mechanical, structural, and functional properties of the cell envelope, which have been so far elusive. Our experiments reveal that cells with a compromised S-layer lattice show a decreased compressive stiffness and elastic modulus. Furthermore, we find that S-layer integrity is required to resist cell turgor under hypotonic conditions. These results present compelling experimental evidence indicating that the S-layers can serve as prokaryotic exoskeletons that support the cell wall in conferring rigidity and mechanical stability to bacterial cells.

Significance Statement Surface layers are a remarkable component of many bacterial cell envelopes, composed of proteins that self-organize into a porous, paracrystalline monolayer that forms a wrapping shell around the cell. Despite their abundance, S-layer function remains poorly understood. Here, we show that in the human and animal pathogen *Bacillus anthracis*, the S-layer forms an important support structure to the cell envelope, that helps it withstand mechanical stresses and osmotic pressure. However, with this comes a vulnerability for molecules or antibodies able to break the ordered S-layer array. A vulnerability we have learned to exploit for *B. anthracis*, and may hold promise for other human pathogens including *Clostridioides difficile*.

## Introduction

The cell envelope is the primary interface between a cell and its environment. Surface layers (S-layers) are 2D paracrystalline protein monolayers commonly found atop prokaryotic cell envelopes. They cover the entire cell surface into a semiporous shell or “armor” (1). The (glyco)-protein(s) that make up bacterial S-layers are noncovalently anchored to the cell wall or outer membrane glycans, and contain a “crystallization” or “assembly” domain that drives self-organization into regular 2D lattices (1). In Archaea, where S-layers are near ubiquitous, they serve as support structures that provide mechanical, thermal, and osmotic stabilization to the cytoplasmic membrane, and demarcate a pericellular compartment known as pseudoperiplasm (2–5). In bacteria, S-layer occurrence is less conserved, estimated to be present in about a third of species. Bacterial S-layer function is often ambiguous and perhaps more pleiotropic, with proposed roles as a molecular sieve for nutrient uptake (6), protection against environmental hazards (7), predators and host responses (8, 9), or as an adhesion factor in the case of pathogenic bacteria (10, 11). Functional studies of bacterial S-layers are made difficult by the fact that they often prove nonessential, sometimes lost or no longer expressed once environmental isolates are cultivated extensively under laboratory conditions (7, 9, 12). Comprising as much as 10%–30% of the cellular protein (1, 13), S-layer production places a high metabolic burden on the cell. What then are the selecting conditions that retain these structures in environmental strains? And does a generalizing function of bacterial S-layers exist?

It has been proposed that analogous to their archaeal counterparts, the primordial role of bacterial S-layers may have been to serve as a cell supporting exoskeleton, and that the pleiotropic functions assigned to individual S-layers are secondary (3). However, experimental evidence in support of such mechanical function is largely lacking, and may be obscured by the fact that the peptidoglycan (PG) cell wall, and in gram-negatives also the outer membrane, have taken up the function as primary cell supporting surface structures in bacteria (14–16). Interestingly though, the PG layer in bacilli lacking an S-layer can be as much as 5 to 10 times the thickness of that in species carrying an S-layer (17, 18) suggesting that when present, the S-layer may take over part of the mechanical support function of the cell wall. In vitro, mechanical studies have shown that high pulling forces of 270 to 300 pN are needed for the removal of hexameric units from the isolated S-layer lattices of *Corinebacterium glutamicum* or *Deinococcus radiodurans (* 19, 20), or have demonstrated the ability of deposited S-layers originating from *Bacillus coagulans* or *B. stereathermophilus* to protect liposomes from thermal, mechanical, or chemical stressors (21–23). In vivo, the mechanical properties and role of bacterial S-layers are yet unclear.

We recently showed that the S-layer of the human and animal pathogen *B. anthracis* plays an important role in cell physiology and infectivity (24). In rich media, the bacterium is known to express one of two mutually exclusive S-layers, comprising Sap or EA1 during exponential or stationary growth phase, respectively (25). Employing Sap depolymerizing nanobodies as S-layer disrupting bio-tools, we were able to induce an acute loss of the *B. anthracis* Sap S-layer integrity, resulting in a drastic remodeling of the cell envelope and a collapse of cells. While noncapsulated *B. anthracis* cells are lined by a smooth, single-layered high contrast boundary corresponding to the Sap S-layer, Nb-treated cells showed irregular nanometric protrusions from the cell surface due to loss of the S-layer boundary (24). These unexpected observations hinted at a possible function of the Sap S-layer as a cell envelope support structure. In this study, we used an array of techniques, including state-of-the-art indentation nanoscopy atomic force microscopy (AFM) (26) to characterize mechanical and phenotypical properties of *B. anthracis* cells lacking their S-layers because of genetic knockout or because of an acute phenotypic disruption by means of S-layer depolymerizing nanobodies.

## Results

### Loss of S-layer integrity alters cell surface texture at micro- and nanoscale

**Fig. 1.**
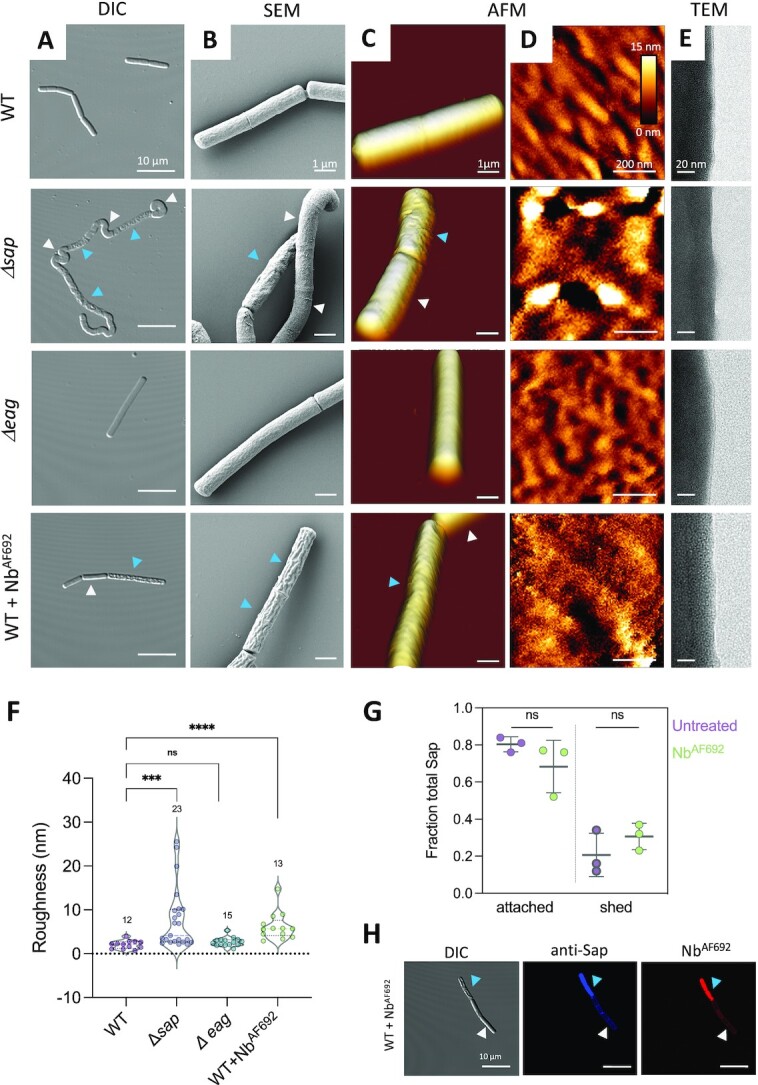
Loss of a crystalline S-layer alters the surface morphology in *B. anthracis* cells. Morphological investigation of *B. anthracis* strains 34F2 (WT), RBA91 (*Δsap*) and SM91 (*Δeag*), or WT cells treated with S-layer depolymerizing nanobody Nb^AF692^ by (A) differential interference contrast (DIC) microscopy, (B) scanning electron microscopy (SEM), and (C) atomic force microscopy (AFM) 3D height and (D) 2D topographic height imaging are presented for a 6 × 6 µm view of whole cells, or 0.7 × 0.7 µm close-up view of the cell surface, respectively, and (E) transmission electron microscope (TEM). Cells lacking a Sap S-layer or cells treated with Nb^AF692^ present a severely altered, wrinkled surface morphology (blue arrows). A minor population have a smooth WT-like surface morphology (white arrows). In TEM, the Sap S-layer is seen as a sharp high contrast demarcation of the cell surface. Images are representative of at least 12 imaged cells from at least 3 independent bacterial cultures. (F) Measured cell surface roughness of individual cells of *B. anthracis* strains 34F2 (WT), RBA91 (*Δsap*) and SM91 (*Δeag*), or WT cells treated with S-layer depolymerizing nanobody Nb^AF692^. Numbers above the violin plots correspond to the number of individual cells probed. Statistical analysis by Mann–Whitney *U* test, with *P* values correspond to ***<0.001, **<0.01, *<0.05, *ns* ≥ 0.05. (G) Fraction of Sap bound to cells or shed in solution for WT or WT + Nb^AF692^ treated cells as determined by anti-Sap western blot. Gray lines and whiskers show mean ± SD of three independent cultures. Statistical analysis by Student *t*-test, with *P* values *ns* ≥ 0.05. (H) DIC and immunofluorescence microscopy of *B. anthracis* strains 34F2 treated with Nb^AF692^ (DyLight-594-labelled) and stained with mouse anti-Sap and DyLight-633-conjugated goat anti-mouse antibodies. Representative cells with smooth or wrinkled morphology are indicated with white and blue arrowhead, respectively.

To investigate the influence of the S-layer on the cell shape and cell envelope structure, we performed a systematic morphological inspection of the noncapsulated *B. anthracis* Sterne strain 34F2 cells with different S-layer compositions, i.e. wildtype (WT); isogenic mutants *Δsap* or *Δeag;* or WT cells treated with a Sap depolymerizing nanobody (Nb^AF692^). Cells were analyzed using light (DIC, Fig. 1A), scanning (SEM, Fig. 1B) and transmission electron (TEM, Fig. 1E), and atomic force (AFM, Fig. 1C and D) microscopies. WT *B. anthracis* form bacilliform cells of approximately 1 by 4 µm, with a smooth and regular surface texture. In contrast, an isogenic mutant lacking the Sap S-layer (*Δsap*) showed curled cells, many of which with a wrinkled appearance (Fig. 1A and B; blue arrows) and an irregular surface topography (Fig. 1C). Strikingly, the *Δsap* mutant shows two morphological populations we here refer to as “smooth” and “wrinkled” (Fig. 1A–C; white and blue arrowheads, respectively). *Δsap* smooth or wrinkled cells have a WT-like or damaged surface appearance, respectively, and correspond to cells that did or did not replace the lacking Sap S-layer with an at least partial EA1 S-layer (24). It is noteworthy that unlike WT, *Δsap* smooth cells showed a curled appearance, suggesting that the lack of Sap and/or the expression of EA1 during exponential phase, alters normal cell growth and cell wall expansion. In the *Δeag* mutant, which holds an intact Sap S-layer during exponential growth, cell morphology and surface texture are similar to that seen for WT (Fig. 1). Cells treated with Nb^AF692^, which induces a rapid depolymerization of the Sap S-layer lattice (24), show a wrinkled phenotype, and a minor populations of smooth cells. Here too, Nb^AF692^ smooth or Nb^AF692^ wrinkled populations correlated with cells that do or do not express at least a partial EA1 S-layer (24).

In addition to these global morphological defects, a lack of the Sap S-layer also altered the cell surface texture (Fig. 1D–F). High-resolution height images obtained on top of the cells show a smooth and undulating surface texture for WT and *Δeag* cells, but reveal a drastic increase in surface roughness upon loss of the Sap S-layer. To better compare the variations among the different S-layers backgrounds, a quantitative analysis of the AFM topographic images was performed to derive the Root Mean Square surface roughness (*Rq*), which provides a measure for the mean peak to valley height in the surface texture (Fig. 1F). WT and *Δeag* cells showed a low roughness with *Rq* values of 2.1 ± 0.9 nm and 2.7 ± 1.0 nm (mean ± SD from *n* = 12 and 15 cells, respectively), determined on 700 × 700 nm² areas. *Δsap* cells showed a broad variation in roughness (*R_q_* = 7.8 ± 7.0 nm, *n* = 23 cells), which could be directly corelated with the smooth and wrinkled morphotypes observed at the microscale. Wrinkled cells, which lack both the EA1 and Sap S-layers, showed a much rougher surface texture, with a *R_q_* of 13.1 ± 6.9 nm (*n* = 11 cells). In *Δsap* smooth cells, the expression of the EA1 S-layer retained a WT-like roughness with *R_q_* of 2.9 ± 0.6 nm (*n* = 12 cells). Also, WT cells treated with Nb^AF692^ were substantially rougher than the untreated cells (WT + Nb^AF692^: *R_q_* = 6.2 ± 3.1 nm from *n* = 13 cells). In TEM, the presence of a crystalline Sap S-layer is seen as a continuous high contrast demarcation of the cell surface of 3 to 4 nm height (Fig. 1E). In vitro, Nb^AF692^ induces a rapid depolymerization of Sap monolayers (24), an activity that is expected to disrupt the lattice properties of the Sap S-layer in vivo, but leave intact the SLH–cell wall interaction and therefore surface association of the Sap monomers (27). Indeed, a quantitative assessment of cell-bound Sap versus the protein in the culture supernatant showed no significant increase in Sap shedding after Nb^AF692^ treatment (Fig. 1 G), suggesting Sap monomers remain bound to the cell surface, probably forming a high-density amorphous layer (24). In agreement with this, immunofluorescence microscopy showed a bright pericellular staining of Sap on wrinkled Nb^AF692^ treated cells (Fig. 1H).

### Perturbation of S-layer integrity induces a softening of *B. anthracis* cells

**Fig. 2.**
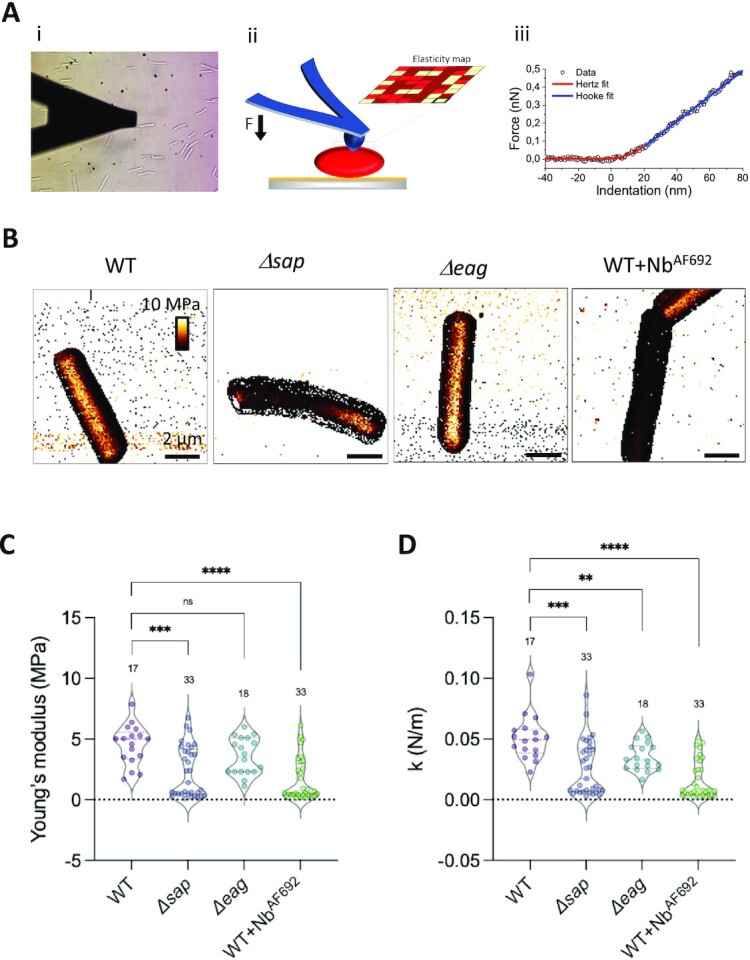
S-layer integrity influences mechanical properties of *B. anthracis* cells. (A) AFM setup used for nanoindentation experiments: (i) a cell of interest is first localized by optical microscopy, (ii) the AFM cantilever is then brought into contact with the cell surface to perform force–distance curves from which the Young’s modulus and the spring constant of the surface area can be extracted (iii). (B) AFM elasticity (Young’s modulus) maps, obtained in quantitative imaging mode, of *B. anthracis* cells presenting different S-layer genetic background or treated with Nb^AF692^. (C and D) Violin plots representing the average cell elasticity (Young’s modulus) and spring constant (*k*), obtained in force volume mode, as a function of the strain. Dashed lines correspond to the data median. Numbers above the violins represent the number of independent cells probed, from at least three independent bacterial cultures. Statistical analysis by Mann–Whitney *U* test, with *P* values correspond to ^****^<0.0001, ***<0.001, **<0.01, *<0.05, *ns* ≥ 0.05.

We next determined the mechanical properties of the various *B. anthracis* cells by means of AFM nanoindentation experiments (26), in which an AFM tip approaches the cell surface, makes contact, and pushes it within a force range where force-induced indentation remains linear, before retracting for a subsequent measurement (Fig. 2A).

**Fig. 3.**
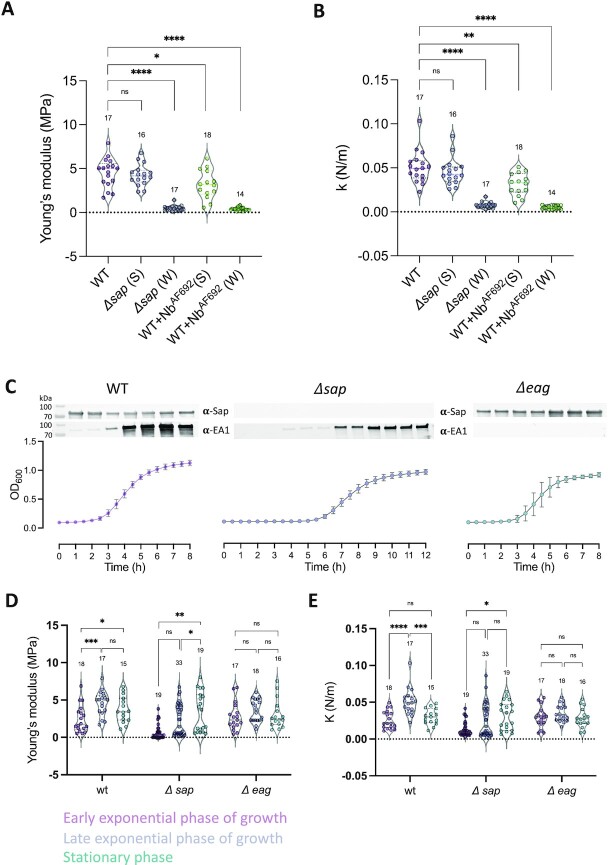
S-layer expression and cell surface nanomechanics of *B. anthracis* at different growth stages. Violin plots quantifying average cell elasticity (Young’s modulus, A) and spring constant (*k*, B), obtained in force volume mode, of *B. anthracis* strains 34F2 (WT; with and without treatment with Nb^AF692^), RBA91 (*Δsap*). Cells were split into smooth (S; minor population) or wrinkled (W; major population) morphology based on microscopic inspection. Dashed lines correspond to the data median. (C) Growth curves of *B. anthracis* strains 34F2 (WT), RBA91 (*Δsap*), and SM91 (*Δeag*) cultured in BHI, and anti-Sap or anti-EA1 western blot analysis of normalized whole cells at the indicated time post inoculation. Average ± SD of three biological replicates. (D) Average cell elasticity (Young’s modulus, D) and spring constant (*k*, E) of *B. anthracis* strains 34F2 (WT), RBA91 (*Δsap*) and SM91 (*Δeag*), and different stages of growth: early and late exponential growth, and stationary phase. Numbers above the violin plots represent the number of independent cells probed, originating from at least three independent bacterial cultures. Statistical analysis by Mann–Whitney *U* test, with *P* values correspond to ^****^<0.0001, ***<0.001, **<0.01, *<0.05, *ns* ≥ 0.05.

Thus, in our experiments, we first mapped the surface elasticity and stiffness of single cells using multiparametric imaging (QI) (Fig. 2A). This mode offers a simultaneous imaging of both topographical and mechanical features of the cell surface (28), images in which contrast and brightness inform on relief and elasticity of the scanned area, respectively. Qualitatively, WT cells and *Δeag*, exhibited a stiffer and less elastic cell envelope than cells with an amorphous or genetically absent S-layer (i.e. WT cells treated with Nb^AF692^ and *Δsap*, respectively) (Fig. 2B). To avoid any edge effects, we then focused on defined areas on top of the cells (250 × 250 nm²) and, using the force–volume (FV) imaging mode, we recorded spatially resolved force curves, which represent the force as a function of the tip-sample distance during the approach-retract cycle (29). The force curves featured two regions—i.e. a nonlinear regime at low forces reflecting surface elasticity followed by a linear regime at higher forces [Fig. 2A (iii)]—from which we assessed the bacterial Young’s modulus, *E*, and the bacterial spring constant, *k*, respectively. A cell’s spring constant is proportional to the turgor pressure and the stiffness of the cell envelope (30, 31). Per cell, the mechanical parameters across the scanned cell surface were then plotted as a histogram (Fig. S1A), from which average elasticity and stiffness were determined (Fig. 2C and D). Of note, because the containment level of our AFM work on *B. anthracis* required the use of chemically fixed cells, we sought to determine the influence of fixation on the mechanical properties of *Bacillus subtilis* cells as a representative nonpathogenic gram-positive model organism. The elastic modulus and bacterial spring constant in fixed or unfixed cells were not significantly different, indicating that our fixation protocol allows the determination of physiologically representative mechanical properties (Fig. S1B and C). Cells possessing an intact, crystalline Sap S-layer (WT and *Δeag* cells) present a Young’s modulus of 4.5 ± 1.6 MPa (mean ± SD from *n* = 17 WT cells) and 3.6 ± 1.5 MPa (*n* = 18 *Δeag* cells) and spring constants of 0.051 ± 0.018 N·m^−1^ and of 0.035 ± 0.011 N·m^−1^ for the respective strains. In sharp contrast, cells genetically lacking the Sap S-layer (*Δsap*) or cells presenting an amorphous Sap S-layer by means of Nb^AF692^-treatment, were more elastic and softer than the untreated WT as shown by the significant shift towards lower *E* (*Δsap*: 2.3 ± 2.1 MPa, *n* = 34; WT + Nb^AF692^: 1.6 ± 1.7 MPa, *n* = 23 cells) and *k* (*Δsap*: 0.026 ± 0.016 N·m^−1^; WT + Nb^AF692^: 0.016 ± 0.015 N·m^−1^). Markedly, the *E* and *k* distribution of *Δsap, Δeag*, and WT + Nb^AF692^ cells showed a bimodal profile, indicative of mechanically distinct populations (Fig. 2C and D). To test if this partitioning of mechanical properties related to micro- and nanoscale morphological features of the cell surface, cells were triaged into the above described “smooth” and “wrinkled” morphotypes using correlative AFM and light microscopy (Fig. 3A and B). *Δsap* smooth cells were found to have *E* and *k* values (*E* = 4.2 ± 1.2 MPa; *k* = 0.045 ± 0.015 N·m^−1^, *n* = 16 cells, pointed with white arrow in Fig. 1A and C) that do not show a significant difference from untreated WT or from *Δeag* cells. On the other hand, *Δsap* wrinkled cells showed a drastic increase in cell elasticity and severe loss of cell stiffness (*E* = 0.5 ± 0.3 MPa; *k* = 0.007 ± 0.003 N·m^−1^, *n* = 17 cells, blue arrows in Fig. 1A and C) (Fig. 3A and B). A similar dichotomy was seen in WT cells treated with Nb^AF692^, where smooth cells show mechanical properties similar to untreated cells (*E* = 3.2 ± 1.6 MPa; *k *= 0.031 ± 0.013 N·m^−1^, *n* = 14 cells) (Fig. 3A and B), and wrinkled cells show a drastic increase in elasticity and loss of stiffness (*E* = 0.4 ± 0.2 MPa; *k* = 0.005 ± 0.002 N·m^−1^, *n* = 18 cells, blue arrows Fig. 1A–C; Fig. 3A and B).

We have previously found that *Δsap* or Nb^AF692^-treated cells with a smooth versus wrinkled surface morphology largely correspond to cells that, respectively, do or do not express at least a partial EA1 S-layer (24), suggesting that EA1 can compensate for structural and mechanical properties of the cell surface endowed by the Sap S-layer. During an early exponential phase, when no EA1 is expressed (Fig. 3C), the full population of *Δsap* cells showed the decreased elastic modulus and reduced bacterial spring constant associated with the *Δsap* wrinkled cells (Fig. 3D and E), whereas in a stationary phase, when at least part of the cells express the EA1 S-layer (Fig. 3C), a second population with WT-like mechanical properties is present (Fig. 3D and E). *Δeag* cells on the other hand, which exclusively express a Sap S-layer both in exponential and stationary phase, showed a single population through cell growth stages, with mechanical properties similar to early exponential stage WT cells (Fig. 3C–E). When *Δeag* cells were treated with Nb^AF692^, all cells showed severe morphological defects, with deep grooves at the cell surface and cell debris around it that prevent the determination of their mechanic properties (Fig. S2A–D). These observations suggest a secondary role of EA1 S-layer, when present, in maintaining the cell surface organization and cell wall mechanics upon loss of Sap S-layer integrity. Of note, we also found the morphological features of *Δsap* cells to change from a curled elongated phenotype in early exponential phase, to a WT-like bacilliform phenotype in stationary phase while *Δeag* cells kept a bacilliform phenotype throughout the different growth phases (Fig. S2E).

### *B. anthracis* S-layer functions as a bacterial exoskeleton

Taken together our morphological and force nanoscopy observations suggest that a crystalline Sap S-layer endows the cell with structural and mechanical stability, a function that can at least in part be taken over by the EA1 S-layer. We have previously shown that in vitro, the Sap assembly domain (Sap^AD^) readily forms S-layer tubules with diameters that approach that of *B. anthracis* cells (24). The Sap lattice itself may therefore have a certain stiffness and act as an elastic crust surrounding the cells. However, Sap^AD^ S-layer tubes readily fragmented when probed using AFM, preventing indentation nanoscopy and suggesting that on itself, the Sap lattice is a brittle structure (Fig. S2F). At the cell surface, however, Sap monomers are attached to the cell wall via the SLH domains (27). To get an observation of the S-layer stiffness when bound to the cell sacculus, we detergent-permeabilized WT cells to remove the contribution of the cell turgor in the measured mechanical properties. The elastic modulus and bacterial spring constant of lysed *B. anthracis* cells were found to be *E* = 1.0 ± 0.5 MPa and *k* = 0.01 ± 0.005 N·m^−1^ (*n* = 19 cells), which are significantly higher than wrinkled *Δsap* or WT + Nb^AF692^ cells (Fig. S2G and H). The residual ∼0.005 N·m^−1^ drop in spring constant in the cells lacking the Sap S-layer versus permeabilized cells is likely to correspond to the stiffness of the crystalline, cell wall-bound Sap S-layer.

**Fig. 4.**
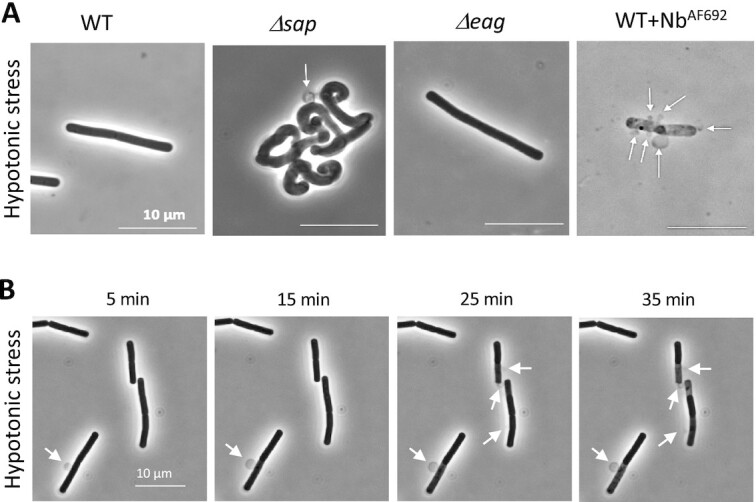
*B. anthracis* S-layer functions as a bacterial exoskeleton. (A) Phase contrast images of *B. anthracis* cells presenting an unperturbed (WT and Δ*eag*), absent (*Δsap*), or amorphous (WT + Nb^AF692^) S-layer placed under hypotonic conditions for 40 min. (B) Timelapse phase contrast images of *B. anthracis* treated with Nb^AF692^ and placed under hypotonic conditions. Cells show localized cytoplasmic membrane bulges (white arrows) of increasing size before resulting in cell lysis.

Finally, we tested if the Sap S-layer also contributes to the ability of the cell wall to withstand turgor pressure. *B. anthracis* cells with WT, *Δsap* or *Δeag* background, or WT cells treated with the Sap S-layer depolymerizing Nb^AF692^ were transferred into pure water and observed by time-lapse light microscopy. Cells with an unperturbed Sap S-layer (WT and *Δeag*) were unaffected by the hypotonic conditions and retained shape and size throughout a 2 h incubation (Fig. 4A). Cells lacking the Sap S-layer (*Δsap*) or displaying a Nb-compromised, i.e. amorphous, Sap S-layer (WT + Nb^AF692^) showed severe membrane blebbing upon transfer to hypotonic conditions, resulting in cell lysis (Fig. 4A and B). These cells retained shape and size, but showed localized blebbing, indicating the Sap S-layer can help protect the cell envelope against local weak spots or breaches in the cell wall sacculus. These evidences further strengthen the hypothesis that *B. anthracis* Sap S-layer plays an essential role in the mechanical stability of cells, likely functioning as a proteinaceous bacterial exoskeleton that supports and possibly helps structuring the cell wall.

## Discussion

The peptidoglycan (PG) cell wall forms a major cell supporting envelope structure that maintains cell shape and can withstand high turgor pressure (14, 32). Genetically or chemically compromised PG synthesis results in morphological deformation and lysis of cells (33, 34). Even localized breaches of the PG network lead to the formation of protruding cytoplasmic membrane bulges or blebs, followed by lysis (35). Accordingly, bacterial cells have evolved elaborate regulatory pathways, multifunctional enzyme complexes, and cytoskeletal structures to orchestrate the localized and timely synthesis, degradation and modification of PG throughout the various stages of the bacterial cell cycle (36). In gram-negative bacteria, the outer membrane forms an additional load-bearing structure in support of a much thinner PG cell wall (16). In addition, many bacteria, both gram-positive and gram-negative, contain a S-layer, a paracrystalline protein monolayer that forms another continuous and pericellular structure. How the synthesis of this structure is orchestrated with cell wall synthesis and whether it forms a substantial component of the mechanical function of the cell envelope are poorly understood. Two recent studies have shown that in *Caulobacter crescentus* and *Clostridium difficile*, the sites for de novo S-layer assembly colocalize with PG synthesis, hinting at a possible direct link between the two processes (37, 38). It is not clear, however, if this colocalized PG and S-layer synthesis is a necessity to ensure cell integrity or a merely consequence of localized growth and division of cells.

Here, we have provided compelling evidence that a *B. anthracis* S-layer confers the cell envelope a mechanical support and stability. Cells with an intact S-layer appear smooth, with a regular undulating surface texture, while cells lacking their S-layer because of genetic knockout or as a result of treatment with lattice disrupting nanobodies show a significative increase in cell roughness, both macroscopically and at the nanoscale (Fig. 1). Strikingly, morphological defects in cells lacking a crystalline S-layer are not restricted to the surface ultrastructure but are also manifested in the whole cell shape. Indeed, cells lacking Sap or presenting an amorphous Sap S-layer due to Nb treatment appear as helical curls rather than the regular bacilliform shape of WT cells (Fig. 1; Fig. S2E). Because a correctly orchestrated PG synthesis is essential for regular cell shape, these observations may hint at a role or influence of the Sap or EA1 S-layers in organizing the sites of cell wall synthesis and remodeling. Previous studies have shown that the Sap and EA1 distribution at the cell surface plays an important role in the positioning of at least some *Bacillus* S-layer-associated proteins or “BSLs” (39–41). BSLs are cell envelope proteins that share the cell wall binding SLH domains found in Sap and EA1, but do not form 2D arrays, and instead represent enzymatic or binding functions participating in different cellular processes such as PG remodeling or bacterial adhesion. Of particular interest, EA1 and Sap S-layer were found to influence the localization of BslO, a BSL with N-acetylglucosaminidase activity found in a surface localized ring at the future cell division plane. Genetic disruption of *sap* resulted in an erroneous pericellular localization of BslO and cell division defects (40).

Using AFM force spectroscopy, we obtain insight in the mechanical properties of S-layers on bacterial cells. These measurements show that cells genetically lacking a Sap S-layer or cells covered in an amorphous S-layer because of Nb-based lattice disruption experience a significant increase in cell elasticity and loss of cell stiffness (Fig. 2). Strikingly, under hypotonic conditions, a lack of crystalline S-layer resulted in severe membrane blebbing and ultimately cell lysis (Fig. 4). Protruding membrane bulges were found to be highly localized, suggestive of local breaches of the PG layer. These observations indicate that, in the absence of a crystalline S-layer, the PG cell wall of *B. anthracis* is insufficient or compromised in its function to withstand cell turgor pressure. The S-layers’ role in cell support may stem from a direct mechanical support of the PG-bound crystalline protein lattice, or a possible function in organizing PG cell wall growth and remodeling. Even so, *Δsap* strains are viable and we have previously found Nb-treated cultures can ultimately overcome the growth attenuation induced by Sap lattice disruption (24). Also, when probing the *Δsap* or Nb^AF692^-treated *B. anthracis* cells by force spectroscopy, we find a striking bimodality in the mechanical and morphological properties, together suggesting that cells can adapt to a loss of crystalline Sap (Fig. 2C and D). This adaptation was at least in part explained by the replacement of the Sap S-layer by an EA1 S-layer [Fig. 3; (24)], but may also include physiological adaptation in the PG synthesis and crosslinking levels. Future investigation of the cross-talk between cell wall synthesis and S-layer integrity will be needed to resolve the molecular mechanisms at play. Furthermore, in pathogenic *B. anthracis* strains poly-γ-d-glutamate chains are covalently anchored to the cell wall and protrude through the semiporous S-layer to cover the cell surface in an antiphagocytic capsule (42). How the capsule traverses the S-layer, or how capsule, S-layer and cell wall production are coordinated are largely unknown.

Taken together, the new experimental evidence provide solid proof for a hitherto unknown and crucial role of the *B. anthracis* S-layer in maintaining cell mechanical stability and participating in cell shape maintenance, thus functioning as a proper bacterial exoskeleton. In Archaea, S-layers are an intricate component of the cell envelope and may predate or replace murein-like polymers as major cell support structures. S-layer are self-organizing semiporous structures composed in most cases of a single protein species. Given this organizational simplicity and in light of our new findings in *B. anthracis*, one may theorize if bacterial S-layers also form an ancestral protein-based cell support and envelope compartmentalizing structure, a function now largely taken over by PG cell walls and an outer membrane.

Finally, we have previously shown that acute disruption of the S-layer lattice by means of nanobodies provides a therapeutic strategy in treating otherwise lethal *B. anthracis* infections (24). We speculate that the role of the S-layer as cell support structure is not exclusive to *B. anthracis*, and that targeting S-layer assembly and integrity can form a viable therapeutic avenue also in other S-layer carrying pathogens.

## Methods

### Bacterial strains growth condition and cell preparation

“WT,” “*Δsap*,” and “*Δeag*”*B. anthracis* as used in this study corresponds to *B. anthracis* Sterne 34F2 (24), and isogenic derivatives RBA91 and SM91, with a spectinomycin-resistance cassette inserted in the *sap* and *eag* gene, respectively (43). *B. subtilis* corresponds to strain 168 (44). Inocula of 0.05 OD_600_ of an overnight preculture were grown at 37°C in Brain Heart Infusion broth (BHI) with shaking and harvested at the late exponential phase of growth (5 h post inoculation for all strains, or 9 h post inoculation for the slower growing *Δsap* strain) or at the indicated time points (early exponential phase of growth: 2 h post inoculation for all strains; stationary phase of growth: 7 h (WT and *Δeag*) and 12 h (*Δsap*) post inoculation; i.e. Fig. 3C–E and Fig. S2E).

Cells subjected to light or atomic force microscopy analysis were fixed during 30 min with PBS supplemented with 4% paraformaldehyde (PFA), with the exception of cells used for the hypotonic condition experiment (Fig. 4), or the *B. subtilis* nonfixed cells (Fig. S1B and C). For SEM analysis, cells were fixed in 2% PFA, 2.5% Glutaraldehyde in 0.1 M Na-Cacodylate buffer pH7.4 during 30 min. For AFM, SEM or light microscopy imaging cells were normalized to an OD_600_ of 1 in PBS or PBS supplemented with 200 µM Nb^AF692^ and incubated during 20 min at room temperature prior to PFA fixing. For the membrane permeabilization experiment (Fig. S2G and H), harvested cells were incubated at RT during 20 min in PBS supplemented with 10% Triton prior to PFA fixation.

For immunofluorescence staining of Sap (Fig. 1G), bacteria were stained with DyLight 594 labelled Nb^AF692^, or mouse antiserum raised against purified recombinant Sap_AD_ followed by incubation with Dylight 633 labelled goat-anti-mouse monoclonal (1:1,000; QL222838; Thermo Scientific) as previously described (24). Prior to imaging cells were spun 2 min at 1500 *g* and taken up in PBS to remove excess buffer, Nb ^AF692^, or anti-mouse secondary antibody.

For the Sap shedding experiment (Fig. 1H), cells were treated with Nb^AF692^ as described above, and the supernatant corresponding to excess of PBS buffer or Nb ^AF692^ supplemented PBS was subjected to TCA precipitation as described elsewhere (45). Sap concentrations in the culture supernatant precipitate or cell pellet were quantified using western blot analysis (see below). Per biological replicate, the % shed or cell-associated Sap was calculated as the ratio of the individual anti-Sap response (supernatant or cell pellet) over the total anti-Sap response (i.e. supernatant + cell pellet).

### S-layer composition by western blot analysis

Cells were grown on BHI broth at 37°C and harvested at the indicated time points (Fig. 3C). Harvested cells were normalized to OD_600_ 1 in 1x Laemmli buffer and incubated during 1 h at 95°C. Whole cell extracts corresponding to 100 µl of cells normalized to OD_600_ 1, were loaded, and separated by Mini-PROTEAN® TGX™ Precast Gels 4%–15% (BioRad) before transfer onto polyvinylidene difluoride (PVDF, mmobilon®-FL PVDF membrane, LI-COR) membrane by western blotting. Blocking, incubation with primary and secondary antibodies and washing of the membrane were done in PBS supplemented with 0.05% Tween-20 (v/v) and 4% (w/v) nonfat dry milk in the case of a blocking step. Membranes were incubated with mouse anti-Sap or anti-EA1 serum (1:1,000) for 2 h (24), washed with PBS, and incubated with Goat anti-mouse IRDye® 800CW (1:20,000; LICOR) for 1 h, washed with PBS, and imaged using an Odyssey M Imager (LICOR). For the shedding experiment (Fig 1G), the western blot protocol differed in that alkaline phosphatase conjugated goat anti-mouse antibody (AQ3562 Sigma-Aldrich) was used as secondary. Blots were developed by incubation with NBT/BCIP (Roche) and imaged and integrated using a GelDoc imager (Biorad). Purified recombinant Sap and EA1 assembly domains (AD) were used as positive controls and Prestained PageRuler (Thermo Fisher Scientific) was used as molecular mass ladder (24).

### Light microscopy

Cells fixed as described above were resuspended upon centrifugation in PBS prior their observation in glass slide and coverslip. DIC microscopy images were acquired with Zeiss LSM 880 airyscan confocal microscope with a magnification of 200x (FIG. 1A and F). In the case of the hypotonic condition experiment (Fig. 4), following the 20 min incubation in PBS or PBS supplemented with 200 µM single Nb^AF692^, cells were resuspended in di-ionized water (MQ) prior their observation in glass slide and coverslip. Cells were observed over 2 h with a Leica Dmi8 microscope and phase contrast images were acquired with an 100x immersion oil objective. Cell blebbing could be observed starting from 5 min post exposition of the cells to the hypotonic environment.

### Electron microscopy

For scanning electron microscopy (SEM) analysis, fixed cells as described above were first washed in 0.1 M Na-Cacodylate buffer pH7.4 and kept at 4 °C over night. Upon removal of Na-Cacodylate, buffer cells were then incubated for 30 min in 2% OsO4 in 0.1 M Na-Cacodylate buffer, pH7.4. Osmicated samples were washed twice in ultrapure water prior to a stepwise ethanol dehydration performed with various concentrations of the latter (50%, 70%, 85%, 100%). A last step of dehydration was performed in hexamethyldisilazane (HMDS) solution (Sigma, Australia). Samples in HMDS were spotted on silicon grids (Ted Pella) and air-dried over night at room temperature. Samples were next coated with 5 nm Platinum (Pt) in a Quorum Q 150T ES sputter coater (Quorum Technologies). The air-dried samples coated with Pt were placed in a Gemini 2 Cross beam 540 Zeiss SEM microscope for imaging at 1.50 kV with a SE2 detector. For TEM analysis, fixed cells were first washed with 0.1 M Na-Cacodylate pH7,4 buffer and then deposited onto a nonglow discharged formvar copper 400 mesh grid (EMS), before staining in 2% uranyl acetate. Negatively stained cells were then imaged using an in-house 120 kV JEM 1400 (JEOL) microscope equipped with a LaB6 filament and CMOS camera (TVIPS TemCam F-416).

### AFM samples preparation

For AFM imaging, bacteria were immobilized on polyethylenimine (PEI)-coated glass coverslips. Those coverslips were incubated overnight, at room temperature, with a 0.2% PEI solution, then rinsed and dried with nitrogen flow before any experiment. A volume of 100 µL of the bacterial diluted suspension, previously vortexed, was then dropped for 1 h on this chemically functionalized substrate and rinsed heavily in PBS bath, to remove any floating bacteria, before AFM measurements.

### AFM multiparametric imaging

AFM images were recorded in PBS at room temperature using a NanoWizard III AFM (JPK Instruments). Imaging was performed in the Quantitative Imaging (QI) mode with soft sharpened silicon nitride cantilevers (MSCT), whose spring constant was initially calibrated with the thermal noise method (k ∼ 0.05 N m^−1^). Images and force curves were recorded at a scan rate of 25 µm s^−1^, an applied force of 0.25 nN and with a resolution of 128 × 128 pixels². Data were analyzed offline using the JPK data processing software. For mechanical analysis, the approach part of the curves was fitted with the Hertz model (Poisson ratio, *ν* = 0.5) over a distance of ∼20 nm and considering a conical tip (opening angle of 17.5°).

### AFM force volume

For mechanical investigation, we privileged the FV mode. Force curves were recorded with a maximum applied force of 0.5 nN and a speed of 1 µm/s. For each cell, a map of 16 × 16 curves was recorded on a 250 × 250 nm² area on top of the cell, in the central region to avoid edge effects. Data were analyzed offline using the JPK data processing software. Young’s modulus of bacteria was extracted from the nonlinear region of the curves, using the Hertz model over ∼20 nm (considering a conical tip with an opening angle of 17.5° and a Poisson ratio of 0.5), while the spring constant was estimated by fitting the following linear part in the curves.

## Supplementary Material

pgac121_Supplemental_Files

## Data Availability

All data are included in the manuscript and/or Supplementary Material.

## Supplementary material

All supplementary files can be found online in the following OSF folder: https://osf.io/cvkyr/?view_only=c88c0431224c4f878750875e599d2983.
