# Supplementary material for: The Bacillus anthracis S-layer is an exoskeleton-like structure that imparts mechanical and osmotic stabilization to the cell wall
Source: PNAS Nexus. 2022 Aug 4;1(4):pgac121. doi: 10.1093/pnasnexus/pgac121 (PMC9802277; doi:10.1093/pnasnexus/pgac121)
Supplement: pgac121_Supplemental_Files [file pgac121_supplemental_files.zip › SI Figure 1.pdf]

SI Figure 1

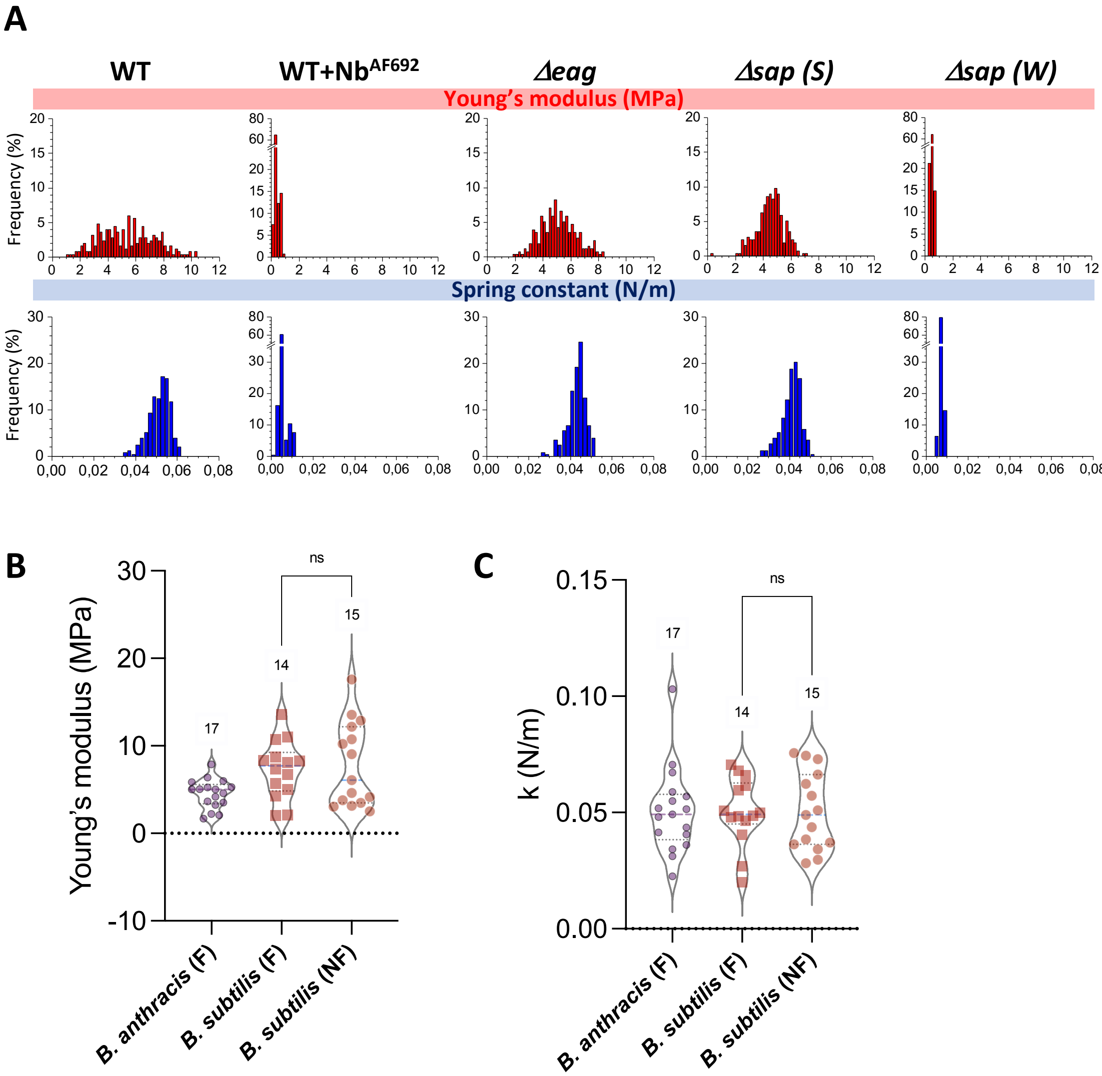

SI Figure 1. Single cell mechanical properties of *B. anthracis* with intact or compromised S-layer. (A) Histograms with mechanical properties (Young's modulus and Spring constant) calculated from force indentation curves obtained across the surface of a representative cell of *B. anthracis* strains 34F2 (WT; with and with Nb^{AF692} treatment), RBA91 (*Δsap*) and SM91 (*Δeag*). For RBA91 histograms are shown for a representative smooth cell (S; minor population) and wrinkled cell (W; main population). Per measured cell, such histograms were used to calculate average Young's modulus and Spring constant shown in Figs. 2, 3 and SI Fig 2B and C. **(B** and **C)** Violin Plots representing the average cell elasticity (Young's modulus) and spring constant (K), obtained in force volume mode, and comparing *B. anthracis* 34F2 and *B. subtilis* cells with (F) or without (NF) prior paraformaldehyde fixation. Dashed line corresponds to the data median. Numbers above the violins represent the number of independent cells probed. Statistical analysis by Mann-Whitney U-test, with P values correspond to ns ≥ 0.05.
