# Supplementary material for: The Bacillus anthracis S-layer is an exoskeleton-like structure that imparts mechanical and osmotic stabilization to the cell wall
Source: PNAS Nexus. 2022 Aug 4;1(4):pgac121. doi: 10.1093/pnasnexus/pgac121 (PMC9802277; doi:10.1093/pnasnexus/pgac121)
Supplement: pgac121_Supplemental_Files [file pgac121_supplemental_files.zip › SI Figure 2.pdf]

SI Figure 2

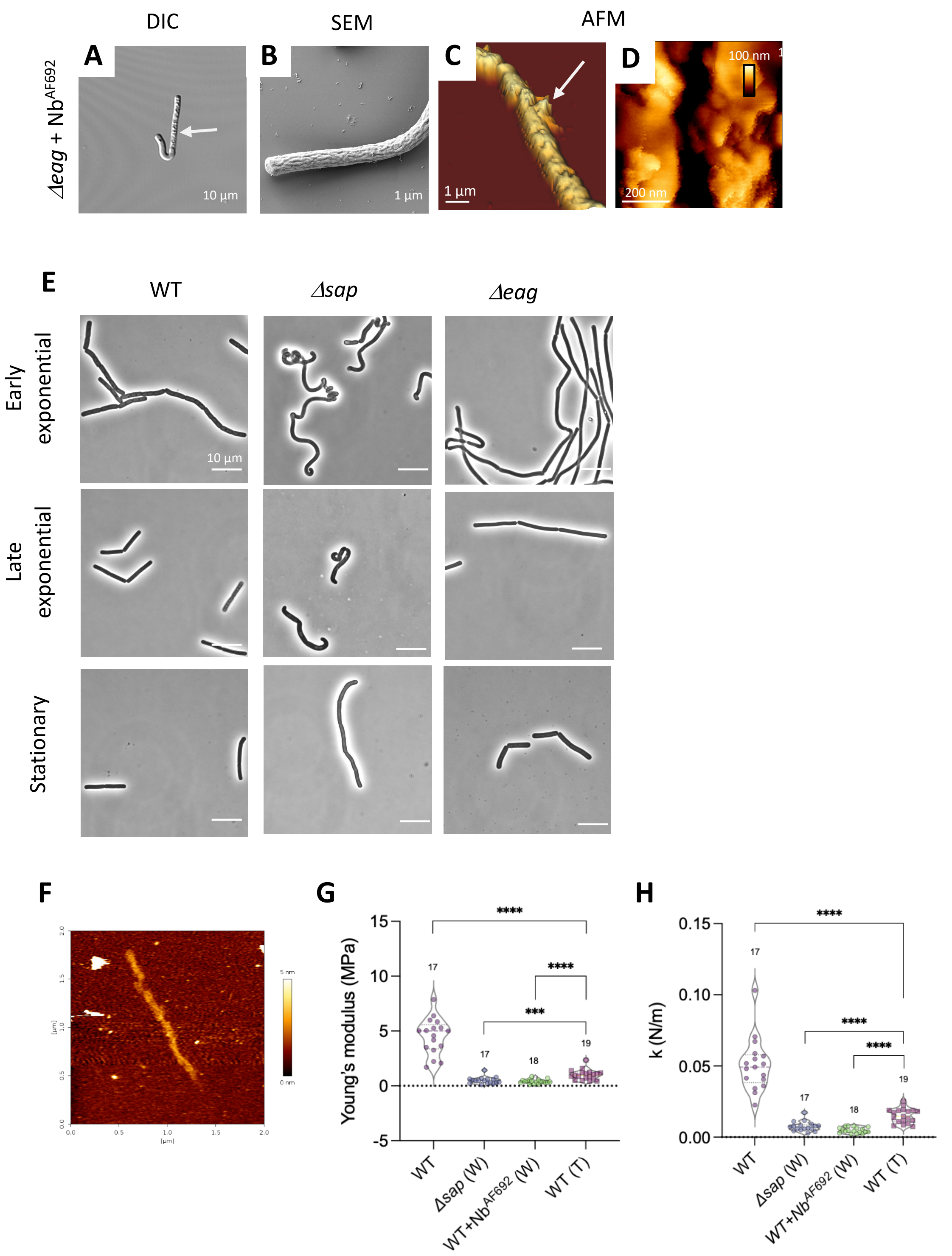

SI Figure 2. Morphology and mechanical properties of *B. anthracis* cells lacking a crystalline Sap S-layer. Imaging of *B. anthracis* SM91 (*Δeag*) cells treated with anti-Sap Nbs^{AF692} using (**A**) Differential interference contrast (DIC) microscopy, (**B**) Scanning Electron Microscopy (SEM),

and (**C**) Atomic force microscopy (AFM) 3D height **(C)** and 2D height images **(D)**. As observed in the WT anti-Sap Nbs^{AF692} treated cells, cells presenting a compromised S-layer because an anti-Sap Nb^{AF692} treatment, present a wrinkled surface (white arrow pointed cells). (**E**) Phase contrast images of *B. anthracis* strains 34F2, RBA91 (*Δsap*) and SM91 (*Δeag*) imaged in different growth phase. In absence of Sap, *B. anthracis* show morphological defects, forming extensively curled cells. (**F**) AFM multiparametric 2D Height image of an *in vitro* S-layer tubule of the purified Sap assembly domain (Sap^{AD}). Even under gentle contact (force set at minimal value to allow imaging), the S-layer tubule is too fragile to be probed without fracturing it. (**G** and **H**) Violin plots representing average cell elasticity (Young's modulus, G) and spring constant (k, H), obtained in force volume mode, for *B. anthracis* strains 34F2 (WT; with and without Nb^{AF692} treatment) and RBA91 (*Δsap*) and *B. anthracis* strains 34F2 permeabilized using triton pretreatment (WT; T). *Δsap* and WT+NB^{AF692} correspond to cells with wrinkled phenotype (W; main population) as derived by prior microscopic imaging. Dashed line corresponds to the data median. Numbers above the violins represent the number of independent cells probed. Statistical analysis by Mann-Whitney U-test, with P values: **** < 0.0001 or ***<0.001.
